# The allometry of cellular DNA and ribosomal gene content among microbes and its use for the assessment of microbiome community structure

**DOI:** 10.1186/s40168-021-01111-z

**Published:** 2021-08-17

**Authors:** Luis Gonzalez-de-Salceda, Ferran Garcia-Pichel

**Affiliations:** Center for Fundamental and Applied Microbiomics and School of Life Sciences, Arizona State University, Tempe, USA

**Keywords:** Bacteria, Archaea, Protists, Fungi, Genomes, Ploidy, Microbiomes, Ribosomal genes

## Abstract

**Background:**

The determination of taxon-specific composition of microbiomes by combining high-throughput sequencing of ribosomal genes with phyloinformatic analyses has become routine in microbiology and allied sciences. Systematic biases to this approach based on the demonstrable variability of ribosomal operon copy number per genome were recognized early. The more recent realization that polyploidy is probably the norm, rather than the exception, among microbes from all domains of life, points to an even larger source bias.

**Results:**

We found that the number of 16S or 18S RNA genes per cell, a combined result of the number of RNA gene loci per genome and ploidy level, follows an allometric power law of cell volume with an exponent of 2/3 across 6 orders of magnitude in small subunit copy number per cell and 9 orders of magnitude in cell size. This stands in contrast to cell DNA content, which follows a power law with an exponent of ¾.

**Conclusion:**

In practical terms, that relationship allows for a single, simple correction for variations in both copy number per genome and ploidy level in ribosomal gene analyses of taxa-specific abundance. In biological terms, it points to the uniqueness of ribosomal gene content among microbial properties that scale with size.

Video Abstract

**Supplementary Information:**

The online version contains supplementary material available at 10.1186/s40168-021-01111-z.

## Background

The rRNA gene approach to microbiome analyses, either based on amplicon or metagenomic sequencing, relies on the tacit assumption that the counts of this marker gene translate into a robust measure or proxy for microbial abundance. However, this assumption is often violated. Sources of error in gene abundance determination can come from analytical procedures such as DNA extraction, PCR amplification, and sequencing itself [1]. But likely as important, systematic biases can be caused by the varying abundance of ribosomal genes in the genomes of microbes [2]. The concern is evident in the dedicated databases that document the variability in ribosomal gene copy number per genome (*R*_*g*_) among microbes [3]. Interestingly, *R*_*g*_ seems to correlate with a microbe’s life history traits, where fast growth is associated with higher values [4–6]. There is also evidence for a certain degree of conservation in *R*_*g*_ within bacterial phylogenetic clades [7]. On this basis, bioinformatic tools have been developed to automatically correct ribosomal gene surveys for *R*_*g*_ [8]. The phylogenetic conservation of *R*_*g*_, however, seems only conspicuous among closely related microbes [9] and can explain only some 10% of its variability in complex, diverse communities [10]. In some eukaryotes like *Saccharomyces cerevisae*,* R*_*g*_ is unstable and can vary widely among strains or individuals [11]. Importantly, such corrections would only lead us to a description of community composition in terms of relative abundance of taxon-specific genome copies. But more useful metrics in microbiome community composition analyses are either cell number [7] (i.e., individuals) or biomass contributions by each taxon. Given the close to 9 orders of magnitude spanned by microbial cell biomass, it can be argued that taxon-specific biomass rather than cell number would be a better descriptor of a taxon’s contribution to a community. However, there are still instances where number of cells would be preferred (for example, to gauge dispersal potential, culturability, or susceptibility to deleterious agents like predators or toxicants). In any case, to translate genome numbers to cell numbers, one needs to take into account the level of ploidy, *P*, the number of copies of the genome present in a cell, where the number of ribosomal operons per cell, *R*_*c*_, is the product *PR*_*g*_. Surprisingly, *P* is not typically taken into account, perhaps under the assumption that most microbes, like *Escherichia coli*, are monoploid [12, 13]. And yet, in bacteria and archaea, *P* varies far more than *R*_*g*_ [12, 14], and most species examined are oligo- or polyploid, with some containing in excess of 200 genomes copies per cell [15]. If one includes unicellular eukaryotes, the variation can be 4 orders of magnitude [16]. Clearly, ploidy constitutes a very important source of bias for community counts in itself [17], affecting estimates from both amplicon sequencing and shot-gun metagenomics. The variable nature of *P* could potentially either compound or diminish the effect of *R*_*g*_ variability in determining a cell’s *R*_c_, as it is not known whether *P* and *R*_*g*_ correlate or vary independently among species; a high *P* could be associated with low *R*_*g*_, and vice-versa. Studies on marine protists intended to estimate biomass from 18S counts have shown that *R*_*c*_ correlated linearly with cell volume (*V*_*c*_) [18] or cell length [19] when plotted on double log scales, indicating an *R*_*c*_ dependence on size.

Here, we posited that perhaps there is constancy among microbes in the need for ribosomal gene content in relation to their cell biomass. In other words, microbial species would be under selection to contain a sufficient but not excessive *R*_*c *_to support the production of their typical cell biomass, *B*_*c*_, so that *R*_*c*_would be proportional to *B*_*c*_. Assuming cell density to be invariant (around 1.008 g ml^−1^) [20], *R*_*c*_ would also be proportional to cell volume (*V*_*c*_).

## Methods

### Dataset

Values for all parameters were gathered or derived from the literature. In place of *B*_*c*_, we used cellular volume, *V*_*c*_, assuming cellular density to be constant (around 1.008 g ml^−1^ [20]). Cell volumes were either taken from reported direct determinations or derived from literature photomicrographs assuming simple formulae for a variety of fitting three-dimensional shapes (i.e., sphere, cylinder) or combinations thereof as given in Table S1 (see Additional file 1). When a range of volume values was available, we used the average. For *R*_*g*_, we used values given in rrnDB [3] for the same species or strain. If they were not available, we used literature values or determined it by examination of the strain’s publicly available genome through BLAST. Ploidy was either taken directly from reported values or estimated if cellular DNA content *and* genome size were known. If *P* was variable within a species or strain, we used the average level of the range given. *R*_*c*_ values were then derived as the product of* P* and *R*_*g*_, although for many protists, *R*_*c*_ was taken directly from experimentally determined values. The annotated input data are gathered in Table S1 (see Additional file 1). The limiting factor to the size of the database was the availability of *P* determinations, which are quite uncommon. In all, we could analyze 107 cases.

### Statistics

Power fits of data were run in Excel as linear regressions of the ln-transformed data pairs using a least-squares model. Statistics are given in Table S2 (see Additional file 2). To test the significance of exponent differences in two separate datasets, we used *T* tests for the slopes of the linear fits.

### Estimation of taxon-specific cell numbers and biovolumes from 16S rRNA counts

In a dataset of rRNA gene taxon-specific frequencies, *F*_*r*_, assigned to *i* taxa whose cell volumes, *V*_*c*_*(i)*, are known, one can directly estimate *R*_*c*_* (i)* from Eq. 1 (see the “Results” section). The relative contribution to number of cells by taxon *i*, *F*_*c*_*(i)*, is computed as:

$${F}_{c }\left(i\right)=\frac{{F}_{r}(i)}{{R}_{c}(i)\sum \frac{{F}_{r}\left(i\right)}{{R}_{c}\left(i\right)}}$$

And the relative contribution to biovolume, *F*_*v*_ *(i)*, as:

$${F}_{v }\left(i\right)=\frac{{F}_{c}(i){ V}_{c}(i)}{\sum {F}_{c}(i){ V}_{c}(i)}$$

If a determination of the absolute abundance of the total copies of the ribosomal gene for all taxa considered in the sample of origin, *R*_*s*_, is available (from qPCR, for example, in units of copies per mass, volume or surface sampled), then absolute taxon-specific assignments *R(i)* can be obtained as the product ${F}_{r}(i)Rs(i).$ From *R(i)*, one can derive absolute values for cells *C(i)* and biovolume *V(i)* attributable to each taxon: *C(i)* = *R(i)/R*_*c*_* (i)* and *V(i)* = *C(i)V*_*c*_*(i)*. The sums $\sum C(i)$ and $\sum V(i)$ estimate the absolute number of cells or biovolume (in µm^3^), respectively, of the entire set of taxa under consideration.

An alternative to using *V*_*c*_*(i)*, if those are not exactly known, is to assign rough discrete size ranges to taxa, *and to* use mean *V*_*c*_ (and *R*_*c*_) values of the range’s maximum and minimum. We found it advisable to set variable-width size ranges in such a way that within-range variation in resulting *R*_*c*_ values was kept moderate. We used the following cell diameter ranges (in µm): 0.2–0.3, 0.3–0.4, 0.4–0.6, 0.6–0.9, 0.9–1.2, 1.2–1.5, 1.5–2.1, 2.1–2.9, 2.9–4.1, 4.1–5.8, 5.8–8.2, 8.2–11.6, and 11.6–16.4. This set provides within-range variation in *R*_*c*_ of less than 8% in all cases, which is smaller than the uncertainty of our estimates for the normalization constant in Eq. 1 of the “Results” section.

## Results

**Fig. 1:**
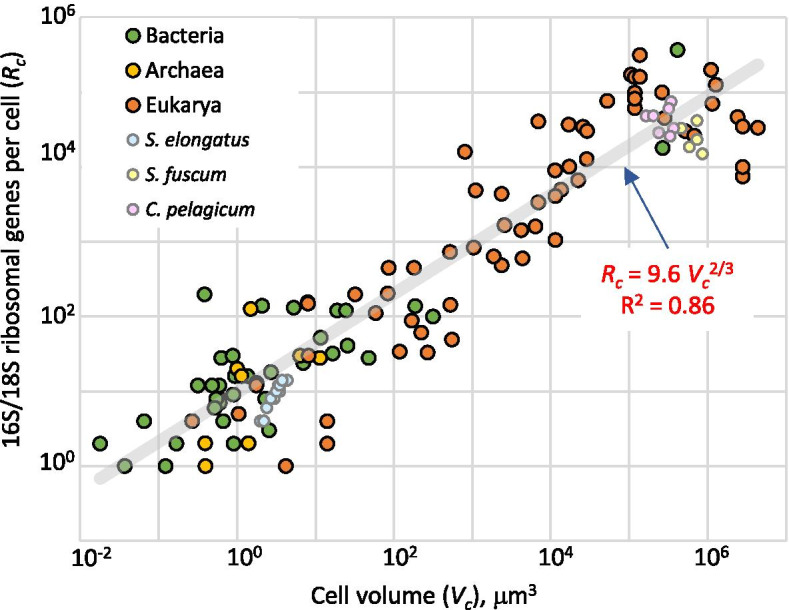
Relationship between cellular ribosomal gene content (*R*_*c*_) and cell volume (*V*_*c*_) in microbes (*n* = 107), plotted as a log/log graph. The grey line is a power fit with the equation displayed in red type (fit statistics are in Table S2, Additional file 2). Data points belonging to eukaryotes are in orange, those for archaea in yellow, and bacteria in green. For three species, we plotted datasets to highlight intraspecies variability: *Synechococcus elongatus* (light blue symbols) [28], *Colozoum pelagicum* (light purple) [19], and *Sphaerozoum fuscum *[19] (light yellow)

Traits that span orders of magnitude are best evaluated as double logarithmic plots, which can be analyzed by power function fits. In this approach, the hypothesis of proportionality between *V*_*c*_ and *R*_*c*_ we posed should have resulted in a power function fit with an exponent close to unity. Our analysis (Fig. 1) readily dispelled that contention. The fit instead revealed that *R*_c_ follows well (*R*^2^ = 0.86) a power function of *V*_*c *_with an exponent significantly lower than unity, and indistinguishable from 2/3 (0.66 ± 0.03; ± SE) across nine orders of magnitude in cell volume. For volumes expressed in µm^3^,

1 $$R_c\:=\:9.58\;v_c^{0.66}\;\cong9.58\;V_c^{2/3}$$

where 9.58 ± 1.21 is the estimated normalization constant. One could envision that the scaling relationship may have been artifactually distorted at the low range of *R*_c_, since it cannot physiologically take values < 1. But a reanalysis of the dataset excluding data pairs with *R*_c_ $\le 2$ did not change the fit significantly in exponent or normalization constant (see Additional file 2). We also tested the hypothesis that exponents for a fit of data pairs from eukaryotes (exponent = 0.72 ± 0.05) vs. prokaryotes (0.62 ± 0.05) could be different, but this did not find strong statistical support in a *T* test comparison (*p* = 0.20).

Equation 1 can be rewritten as a function of linear cell dimensions using a spherical-equivalent cell diameter, ${D}_{c }^{0}=2 \sqrt[3]{\frac{3 {V}_{c}}{4\pi }}$ so that.

2 $$R_c\:=\:6.25\;\left(D_c^0\right)^{1.98}\;\cong\;6.25\;\left(D_c^0\right)^2$$

Thus, *R*_c_ scales generally not with the volume but with the surface area of a microbial cell, which for the purpose of this study means that the bias associated with ribosomal gene counts will be size-dependent regardless of our choice of abundance estimator. Ribosomal counts will overestimate large-celled microbes over small-celled ones if one is interested in number of cells, the bias increasing with the square of linear cell size (Eq. 2), a prediction that finds experimental support for specific cases in the literature [21]. In terms of biomass, ribosomal counts will underestimate the contribution of large microorganisms, the bias increasing with the 2/3 power of cellular biovolume (Eq. 1). Whichever the desired measure of abundance, however, the explicit relationship in Fig. 1 provides a means for bias correction in tallies of ribosomal genes, as long as cell biovolume is known from ancillary data for the taxa detected in the microbiome of interest. The correction requires knowledge of neither *P* nor *R*_g_.

**Fig. 2:**
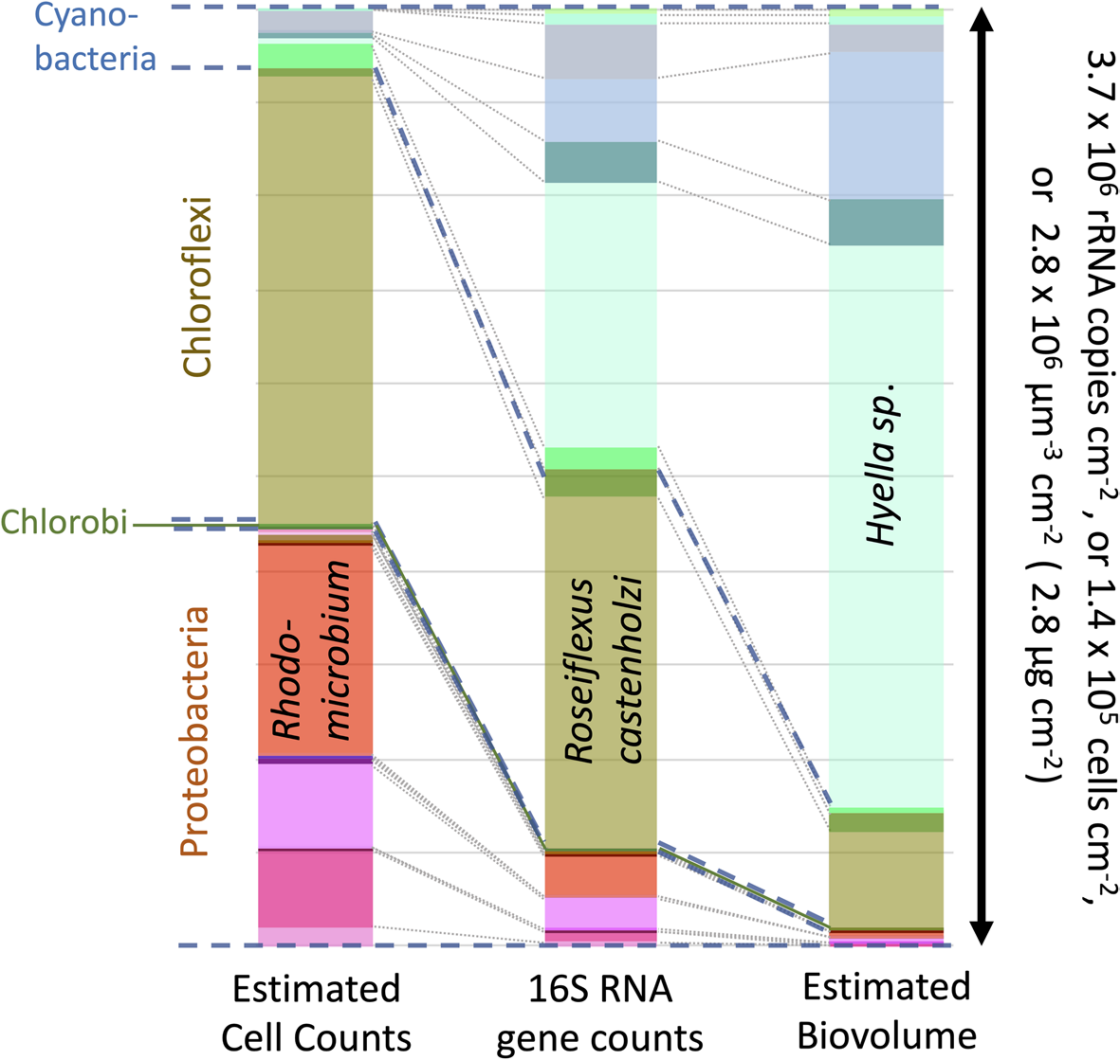
Estimation of microbial community structure based on experimental ribosomal counts (central column), estimated cell number (left column) and estimated biovolume (right column) in a single, exemplary dataset using allometric corrections based on Eq. 1. The dataset is from Roush et al. [22] and includes the subset of taxonomically assignable phototrophic bacteria from an endolithic microbiome on coastal marine carbonate rocks. Only three exemplary phototrophs are labeled, but full, taxonomically explicit distributional data are in Table S3 (see Additional file 3). For ease of comparison, results are graphically presented as relative frequencies, but absolute scales of areal abundance are indicated on the arrow to the right

A procedural explanation is given under the “Methods” section, and we provide an example application in Fig. 2 using a dataset of phototrophic bacteria from endolithic microbiomes within intertidal hard carbonate rocks [22], responsible for their micritization and bioerosion [23], and useful here because typical cell volumes could be assigned to all taxa. The differential outcomes are obvious: 16S rRNA counts of large-celled cyanobacterial genera severely underestimate their contribution to biomass but overestimate their contribution in terms of number of cells (see for example, *Hyella *sp.). The opposite is true for alphaproteobacterial phototrophs (see for example *Rhodomicrobium *sp.), most of which are small-celled [24]. The distortion is less intense for the Chloroflexi, with intermediate cell size (see *Roseiflexus castenholzii*, for example).

We have presented the issue of bias having in mind relative abundance tallies of microbiome members, but proportional tallies have methodological constraints in themselves, because the individual proportions must add up to 1, and thus the relative abundances of taxa are necessarily not independent of each other. There is clear evidence of severely diverging analytical outcomes when both relative and absolute abundance are compared in the same datasets [25, 26]. Commonly, relative proportions or taxa-specific ribosomal copies are converted to absolute abundances with parallel quantification of rRNA gene copies by qPCR, either total copies in the community analyzed or those of particular taxa [16]. We note here that, in view of our results, the latter would require allometric correction, whereas the former would not (as done in the dataset presented in Fig. 2) and is thus a preferable approach. However, we also note that the total number of ribosomal gene copies in a sample is not a good absolute measure of the combined microbial biomass or number of cells present for comparisons among samples, as it will be dependent on their inherent cell-size distribution. Hence, comparisons among samples will only be meaningful if carried out after conversion to biomass or cell numbers, unless the microbial composition of the samples is unchanged.

## Discussion

The procedure outlined here requires knowledge of morphological metadata in addition to sequencing counts for each taxon. Unfortunately, cell volume data are not readily available for many taxa, at least in a compiled format, and requires intensive literature searches. In its absence, and as an approximation, using a few discrete cell-size classes instead of exact values yields useful corrected distributions (see Figure S1 in Additional file 4). Yet, an effort to bring microbial size data into a consolidated platform would be desirable in that it would enable the processing of large datasets in an automated, more manageable way.

An additional factor to take into account is the substantial data spread around the fit leading to Eq. 1, which can limit the precision of the correction. An expanded dataset should improve predictive accuracy and perhaps even precision, but some inherent limitations are also at play. *P* can vary in a single strain with cell cycle [15] and growth conditions [27]. We have included the range of intraspecies variability on the *V*_*c*_*/R*_*c*_ space in Fig. 1 for the cases of a single strain of *Synechococcus elongatus*, and of single cells from natural populations of *Sphaerozoum fuscum* and *Colozoum pelagicum*. They suggest that a significant proportion of spread can be attributed to biological intraspecies variability, tempering the prospects for improvement with eventually extended datasets. Studies on *Synechococcus elongatus* [28–30] and *Saccharomyces cerevisae* [31] *point to a regulatory interdependency of* *P* with cell size, indicating that natural variations in ploidy may be met by commensurate variation in volume, making this much less of a problem. Additionally, because the data used here were arrived at through several approaches, a dedicated survey based on more consistent analytical procedures may result in tighter fits. Finally, part of the variability detected may have been due to neglecting contributions of organelle ribosomal genes in protists. This is expected to be negligible for large-celled eukaryotes, but perhaps not so much for the smallest of them, in which organelles take up a larger portion of their cell volume. Indeed, some of these pico-eukaryotes contribute disproportionately (by defect in *Rc*) to the regression’s sum of squares and may have contributed to the somewhat higher exponent in the eukaryote-only fit (Additional file 2). In support of this contention, a re-analysis excluding eukaryotes with *V*_*c*_ < 20 µm^3^ yields an exponent (0.66 ± 0.06; *R*^2^ = 0.68), more in line with that of Eq. 1, showing no trace of statistical difference (*T* test, *p* = 0.68) with that of the prokaryote-only fit (Additional file 2). While the dataset does not allow us to differentiate between bacteria and archaea because of the low number of archaeal cases in it, given the substantial biological differentiation between bacteria and archaea, it may be an interesting future exercise.

The preceding discussion on uncertainty in the correction approach should not be taken as grounds for inaction, given that the range of variation in *V*_c_ far exceeds that traceable to deviations from the fit, not only among microbes at large, but also in specific settings, and the spectra of microbial size distribution in microbiomes seems to be dynamic. For example, the range of *V*_*c*_ of typical bacterioplankton (excluding phototrophs) in seawater spans 3 orders of magnitude and its spectrum can be modified significantly by factors like grazing [32]. Considering photosynthetic plankton would likely add another 4–5 orders of magnitude in *V*_*c*_, and the size spectrum of this group is also affected by environmental parameters [33]. In the human gut microbiome, our initial assessments show that microbiome typical bacteria span over at least 4 orders of magnitude in volume.

Beyond the pragmatic uses for community composition corrections, we see it as unlikely that the apparent scaling relationship with cell surface area has no biological meaning. It is tempting to speculate that *R*_c_ scales with size to maintain an increasing protein content need. Indeed, protein content scales as a function of cell volume with a similar exponent of 0.70 ± 0.06 (*R*^2^ = 0.87; 95% CI 0.64–0.75) [34]. Because the CI of the exponent for protein content per cell and that for *R*_c_ in the fit of Fig. 1 [0.72 and 0.61; see (Additional file 2)] overlap, the possibility of a connection to cellular need for proteins cannot be rejected solely on this basis. Indeed, in *Synechococcus elongatus* in the laboratory, protein content and *P* (hence also *R*_c_) strongly co-vary with cell volume [30].

**Fig. 3:**
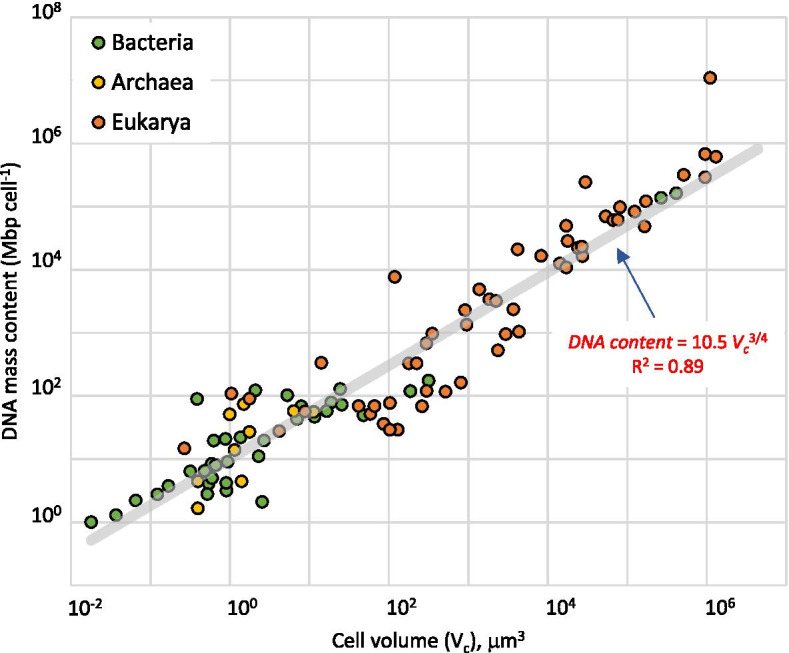
DNA content scales with cell volume as a power function with an exponent of ¾. Entries are from a subset of those in Table S1 (*n* = 60, see Additional file 1), and determinations by Shuter et al. [35] (*n* = 39). Orange points belong to eukaryotic microbes, yellow points belong to archaea, and green points to bacteria. Full statistics for the fit (in red type) are given in Table S2 (Additional file 2)

Alternatively, and perhaps more trivially, the scaling relationship of *R*_c_ with *V*_*c*_ may simply be a reflection of the size scaling of DNA content per cell. In other words, ribosomal genes would follow the trends of DNA content as a whole, just like any other universal gene would. The allometric relationship between DNA content and cell size, however, has not been addressed in the literature or has been addressed incorrectly by neglecting ploidy [34, 35]. We know that genome size scales among bacteria with reported exponents between 0.21 (*R*^2^ = 0.60) [34] and 0.35 (*R*^2^ = 0.45) [36]. In our dataset, which includes eukaryotes, it does so with an exponent of 0.18 (*R*^2^ = 0.34; see Additional file 5). Even when these coefficients of correlation are rather poor, genome size clearly increases much more weakly with *V*_*c*_ than does *R*_c_. Again, this does not take into account *P* variations to yield actual DNA content per cell; it is the size of one copy of the genome. A portion of our dataset can be used to explore the scaling of DNA content per cell for prokaryotes (*n* = 60). To this subset, we can add the measured or slightly derived values reported by Shuter et al. [35] (*n* = 39), excluding those that relied on assumptions of monoploidy. This combined set yields a power scaling fit with *R*^2^ = 0.89 and estimated exponent of ¾ (0.75 ± 0.03; Fig. 3).

The difference in scaling exponent between genome size and cell DNA content (0.18–035 vs. 0.75) gauges the importance of *P*. In fact, in our dataset, *P* seems to scale with *V*_*c*_ as a power law with an exponent of 0.54 (*R*^2^ = 0.69; Additional file 6). This is consistent with the fact that the product of genome size and ploidy yields the cell DNA content, as the exponents of the multipliers (0.18 and 0.54, respectively) roughly add up to the estimated exponent of the product (0.75). That the exponents for DNA content per cell (3/4) and *R*_c_ (2/3) are significantly different (*T* test, *p* = 0.02), speaks for respective mechanistic drivers that are fundamentally decoupled. In fact, most known allometric laws found in nature scale with exponents that are simple multiples of 1/4 [37]. It would seem that ribosomal genes are, in that sense, unique.

## Conclusions

The results presented here uncover surprising basic rules on the composition of microbes, rules that ties them all together, and that far from being self-evident, pose an intellectual challenge to elucidate. In practical terms, this discovery also provides a rather simple approach to deal with biases affecting the use of current omics methodologies for the assessment of microbiome composition, which, given their extensive use in many areas of microbiology and allied sciences, has a large potential for applicability.

## Supplementary Information

**Additional file 1: Table S1**. Taxon-specific values for primary variables (cell volume, ribosomal gene copies per cell, cellular DNA content and ploidy) as well as source variables (cell shape, cell axial dimension, ribosomal gene copies per genome and genome size) as used in the analyses presented in Fig.1

**Additional file 2:Table S2**. Statistics and estimated parameters for power fits against Vc.

**Additional file 3:Table S3**. Explicit dataset used in Fig. ure 2. Original 16S rRNA gene amplicon sequencing data, taxonomic assignments, and qPCR 16S rRNA gene quantifications are from Roush et al. (2020). Estimations of taxon-specific cell number and taxon-specific biovolume according to Materials and Methods.

**Additional file 4: Figure S1**. Differences in allometric estimation of microbial community structure as cell number or biovolume from 16S rRNA gene counts in the dataset of Fig.  2 by either assigning measured cell volume values to taxa or by assigning taxa to a set of discrete size ranges. Left: stack bar graphs for relative proportions of taxa. Right: frequency histograms for taxa-specific percentual differences between the two approaches. 

**Additional file 5:Figure S2**. Relationship between genome size and cell volume (Vc) in microbes (n = 56), plotted as a log/log graph. The grey line is a power fit with the equation displayed in red type (fit statistics are in Suppl. Table 2). Datapoints belonging to eukaryotes are in orange, those for prokaryotes in green.

**Additional file 6:Figure S3**. Relationship between ploidy (P) and cell volume (Vc) in microbes (n = 56), plotted as a log/log graph. The grey line is a power fit with the equation displayed in red type (fit statistics are in Suppl. Table 2). Datapoints belonging to eukaryotes are in orange, those for prokaryotes in green.

## Data Availability

All data generated or analyzed during this study are included in this published article and its supplementary information files.

## Abbreviations

*R*_g_: Ribosomal gene copy number per genome
*R*_c_: Ribosomal gene copy number per cell
*R*_s_: Total copies of the ribosomal gene for all taxa considered in a sample
*V*_c_: Cell volume
*B*_c_: Cell biomass
*D*_c_: Cell diameter
P: Ploidy

## References

[1] Brooks JP (2015). The truth about metagenomics: quantifying and counteracting bias in 16S rRNA studies. BMC Microbiol.

[2] Lavrinienko A, Jernfors T, Koskimäki JJ, Pirttilä AM, Watts PC. Does Intraspecific Variation in rDNA Copy Number Affect Analysis of Microbial Communities?. Trends Microbiol. 2021;29(1):19–27. 10.1016/j.tim.2020.05.019. 10.1016/j.tim.2020.05.019 32593503

[3] Stoddard SF, Smith BJ, Hein R, Roller BRK, Schmidt TM (2014). rrnDB: improved tools for interpreting rRNA gene abundance in bacteria and archaea and a new foundation for future development. Nucleic Acids Res.

[4] Klappenbach JA, Dunbar JM, Schmidt TM (2000). rRNA operon copy number reflects ecological strategies of bacteria. Appl Environ Microbiol.

[5] Roller BR, Stoddard SF, Schmidt TM (2016). Exploiting rRNA operon copy number to investigate bacterial reproductive strategies. Nat Microbiol.

[6] Vieira-Silva S, Rocha EP. The systemic imprint of growth and its uses in ecological (meta)genomics. PLoS Genet. 2010;6(1):e1000808. 10.1371/journal.pgen.1000808. 10.1371/journal.pgen.1000808 PMC2797632 20090831

[7] Kembel SW, Wu M, Eisen JA, Green JL. Incorporating 16S gene copy number information improves estimates of microbial diversity and abundance. PLoS Comput Biol. 2012;8(10):e1002743. 10.1371/journal.pcbi.1002743. 10.1371/journal.pcbi.1002743 PMC3486904 23133348

[8] Angly FE (2014). CopyRighter: a rapid tool for improving the accuracy of microbial community profiles through lineage-specific gene copy number correction. Microbiome.

[9] Lofgren LA (2019). Genome-based estimates of fungal rDNA copy number variation across phylogenetic scales and ecological lifestyles. Mol Ecol.

[10] Louca S, Doebeli M, Parfrey LW (2018). Correcting for 16S rRNA gene copy numbers in microbiome surveys remains an unsolved problem. Microbiome.

[11] Kwan EX, Wang XS, Amemiya HM, Brewer BJ, Raghuraman MK. rDNA Copy Number Variants Are Frequent Passenger Mutations in Saccharomyces cerevisiae Deletion Collections and de Novo Transformants. G3 (Bethesda, Md.). 2016;6(9):2829–38. 10.1534/g3.116.030296. 10.1534/g3.116.030296 PMC5015940 27449518

[12] Pecoraro V, Zerulla K, Lange C, Soppa J. Quantification of ploidy in proteobacteria revealed the existence of monoploid, (mero-)oligoploid and polyploid species. PloS one. 2011;6(1):e16392. 10.1371/journal.pone.0016392. 10.1371/journal.pone.0016392 PMC3031548 21305010

[13] Trun NJ. Genome Ploidy. In: de Bruijn FJ, Lupski JR, Weinstock GM. (eds) Bacterial Genomes. Boston: Springer; 1998. 10.1007/978-1-4615-6369-3_9.

[14] Soppa J (2014). Polyploidy in archaea and bacteria: about desiccation resistance, giant cell size, long-term survival, enforcement by a eukaryotic host and additional aspects. J Mol Microbiol Biotechnol.

[15] Maldonado R, Jiménez J, Casadesús J (1994). Changes of ploidy during the Azotobacter vinelandii growth cycle. J Bacteriol.

[16] Bonk F, Popp D, Harms H, Centler F (2018). PCR-based quantification of taxa-specific abundances in microbial communities: quantifying and avoiding common pitfalls. J Microbiol Methods.

[17] Soppa J (2017). Polyploidy and community structure Nature microbiology.

[18] Godhe A (2008). Quantification of diatom and dinoflagellate biomasses in coastal marine seawater samples by real-time PCR. Appl Environ Microbiol.

[19] Biard T (2017). Biogeography and diversity of Collodaria (Radiolaria) in the global ocean. ISME J.

[20] Guerrero R, Pedrós-Alió C, Schmidt TM, Mas J (1985). A survey of buoyant density of microorganisms in pure cultures and natural samples. Microbiologia (Madrid, Spain).

[21] Jasso-Selles DE, De Martini F, Velenovsky JF, IV Mee ED, Montoya SJ, Hileman JT, Garcia MD, Su NY, Chouvenc T, Gile GH. The Complete Protist Symbiont Communities of *Coptotermes formosanus* and *Coptotermes gestroi*: Morphological and Molecular Characterization of Five New Species. J Eukaryot Microbiol. 2020;67:626-41. 10.1111/jeu.12815. 10.1111/jeu.12815 32603489

[22] Roush D, Garcia-Pichel F (2020). Succession and colonization dynamics of endolithic phototrophs within intertidal carbonates. Microorganisms.

[23] Chacón E, Berrendero E, Pichel FG (2006). Biogeological signatures of microboring cyanobacterial communities in marine carbonates from Cabo Rojo. Puerto Rico Sedimentary Geology.

[24] Overmann J, Garcia-Pichel F (2006). The phototrophic way of life. The prokaryotes.

[25] Props R (2017). Absolute quantification of microbial taxon abundances. ISME J.

[26] Fernandes VM (2018). Exposure to predicted precipitation patterns decreases population size and alters community structure of cyanobacteria in biological soil crusts from the Chihuahuan Desert. Environ Microbiol.

[27] Paranjape SS, Shashidhar R. The ploidy of Vibrio cholerae is variable and is influenced by growth phase and nutrient levels. FEMS Microbiol Lett. 2017;364(19):10.1093/femsle/fnx190. 10.1093/femsle/fnx190. 10.1093/femsle/fnx190 28961807

[28] Ohbayashi R, Nakamachi A, Hatakeyama TS, Watanabe S, Kanesaki Y, Chibazakura T, Yoshikawa H, Miyagishima SY. Coordination of Polyploid Chromosome Replication with Cell Size and Growth in a Cyanobacterium. mBio. 2019;10(2):e00510–19. 10.1128/mBio.00510-19. 10.1128/mBio.00510-19 PMC6478999 31015323

[29] X-y Z, O’Shea EK (2017). Cyanobacteria maintain constant protein concentration despite genome copy-number variation. Cell Rep.

[30] Chen AH, Afonso B, Silver PA, Savage DF. Spatial and temporal organization of chromosome duplication and segregation in the cyanobacterium Synechococcus elongatus PCC 7942. PloS one. 2012;7(10):e47837. 10.1371/journal.pone.0047837. 10.1371/journal.pone.0047837 PMC3480399 23112856

[31] Mundkur BD (1953). Interphase nuclei and cell sizes in a polyploid series of Saccharomyces. Experientia.

[32] Andersson A, Larsson U, Hagström Å. Size-selective grazing by a microflagellate on pelagic bacteria. Mar Ecol Prog Ser. 1986;33:51–57.

[33] Marañón E (2001). Patterns of phytoplankton size structure and productivity in contrasting open-ocean environments. Mar Ecol Prog Ser.

[34] Kempes CP, Wang L, Amend JP, Doyle J, Hoehler T (2016). Evolutionary tradeoffs in cellular composition across diverse bacteria. ISME J.

[35] Shuter BJ, Thomas J, Taylor WD, Zimmerman AM (1983). Phenotypic correlates of genomic DNA content in unicellular eukaryotes and other cells. Am Nat.

[36] DeLong JP, Okie JG, Moses ME, Sibly RM, Brown JH (2010). Shifts in metabolic scaling, production, and efficiency across major evolutionary transitions of life. Proc Natl Acad Sci.

[37] West GB, Woodruff WH, Brown JH (2002). Allometric scaling of metabolic rate from molecules and mitochondria to cells and mammals. Proc Natl Acad Sci.

