# Supplementary material for: The allometry of cellular DNA and ribosomal gene content among microbes and its use for the assessment of microbiome community structure
Source: Microbiome. 2021 Aug 17;9:173. doi: 10.1186/s40168-021-01111-z (PMC8371883; doi:10.1186/s40168-021-01111-z)
Supplement: Supplementary file 3 — Additional file 2:Table S2. Statistics and estimated parameters for power fits against Vc. [file 40168_2021_1111_MOESM3_ESM.docx]

**Table S2.** Statistics and estimated parameters for power fits against *V_c_*

| **Variable** | **Exponent (SE; 95% CI)** | **Norm. constant (SE)** | **n** | **R^2^** |
| --- | --- | --- | --- | --- |
| *R_c_* | 0.659 (0.03; 0.71-0.61) | 9.58 (1.21) | 106 | 0.86 |
| *R_c_* (prokaryotes) | 0.623 (0.05; 0.73-0.51) | 11.26 (1.20) | 44 | 0.75 |
| *R_c_* (eukaryotes) | 0.716 (0.05; 0.81-0.62) | 5.44 (1.56) | 61 | 0.79 |
| *R_c_* (>$2$) | 0.649 (0.03; 0.70-0.54) | 10.68 (1.24) | 99 | 0.85 |
| *R_c_* (eukaryotes; >20) | 0.658 (0.06; 0.78-0.54) | 10. 33 (1.87) | 53 | 0.68 |
| Cell DNA content | 0.746 (0.03; 0.80-0.69) | 10.46 (1.19) | 98 | 0.89 |
| *Genome size* | 0.184 (0.03; 0.25-0.12) | 3.94 (1.16) | 60 | 0.34 |
| *P* | 0.538 (0.05; 0.63-0.45) | 3.12 (1.24) | 61 | 0.69 |
