# Supplementary material for: The allometry of cellular DNA and ribosomal gene content among microbes and its use for the assessment of microbiome community structure
Source: Microbiome. 2021 Aug 17;9:173. doi: 10.1186/s40168-021-01111-z (PMC8371883; doi:10.1186/s40168-021-01111-z)
Supplement: Supplementary file 5 — Additional file 4: Figure S1. Differences in allometric estimation of microbial community structure as cell number or biovolume from 16S rRNA gene counts in the dataset of Fig. 2 by either assigning measured cell volume values to taxa or by assigning taxa to a set of discrete size ranges. Left: stack bar graphs for relative proportions of taxa. Right: frequency histograms for taxa-specific percentual differences between the two approaches. [file 40168_2021_1111_MOESM5_ESM.docx]

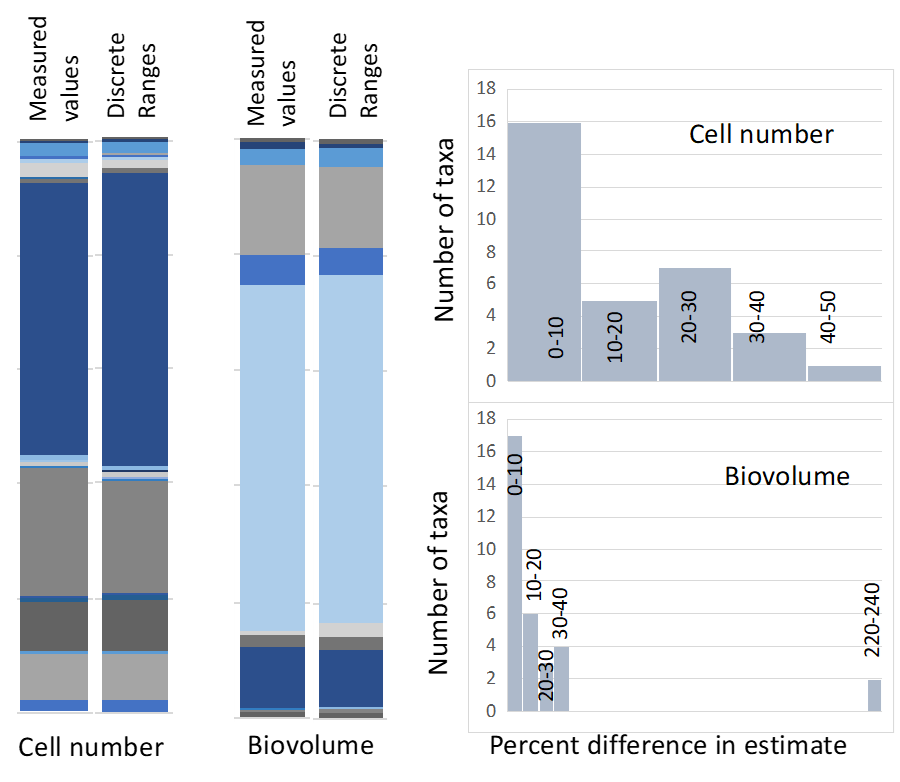


**Fig. S1.** Differences in allometric estimation of microbial community structure as cell number or biovolume from 16S rRNA gene counts in the dataset of Fig. 2 by either assigning measured cell volume values to taxa or by assigning taxa to a set of discrete size ranges. Left: stack bar graphs for relative proportions of taxa. Right: frequency histograms for taxa-specific percentual differences between the two approaches.
