# Supplementary material for: The allometry of cellular DNA and ribosomal gene content among microbes and its use for the assessment of microbiome community structure
Source: Microbiome. 2021 Aug 17;9:173. doi: 10.1186/s40168-021-01111-z (PMC8371883; doi:10.1186/s40168-021-01111-z)
Supplement: Supplementary file 7 — Additional file 6:Figure S3. Relationship between ploidy (P) and cell volume (Vc) in microbes (n = 56), plotted as a log/log graph. The grey line is a power fit with the equation displayed in red type (fit statistics are in Suppl. Table 2). Datapoints belonging to eukaryotes are in orange, those for prokaryotes in green. [file 40168_2021_1111_MOESM7_ESM.docx]

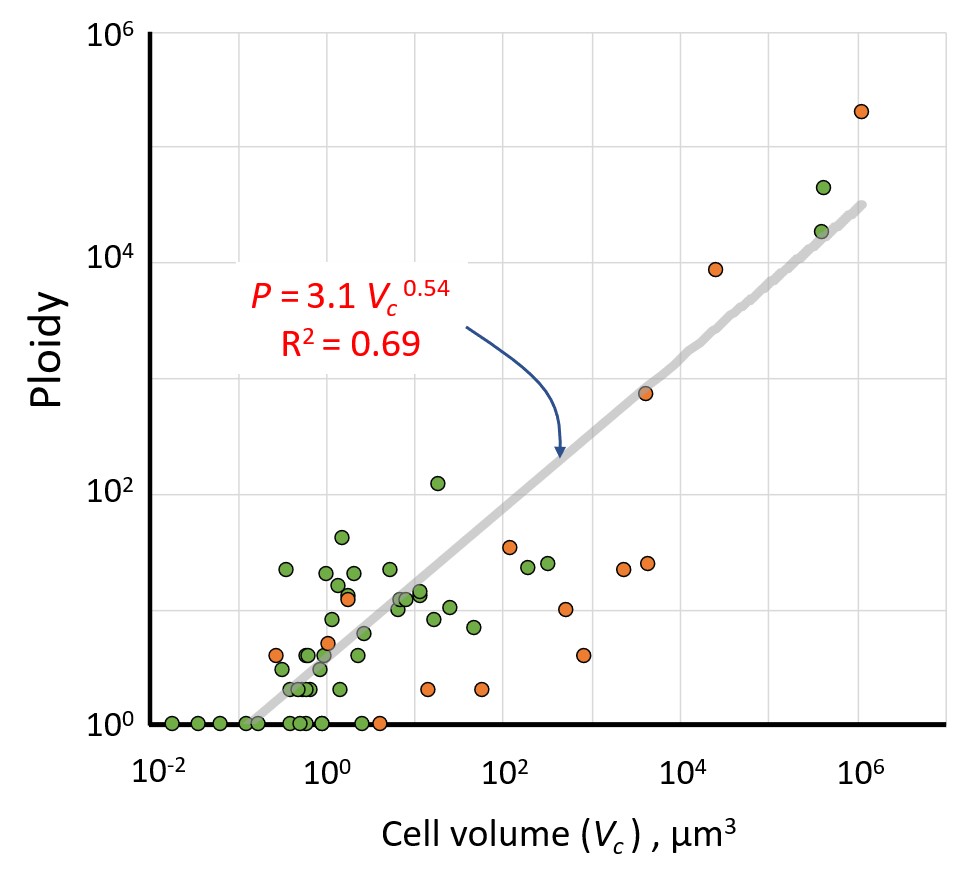


**Fig. S3.** Relationship between ploidy (*P*) and cell volume (*V_c_*) in microbes (n = 56), plotted as a log/log graph. The grey line is a power fit with the equation displayed in red type (fit statistics are in Suppl. Table 2). Datapoints belonging to eukaryotes are in orange, those for prokaryotes in green.
